# Bone Marrow-Derived Mononuclear Cell Therapy in Papain-Induced Experimental Pulmonary Emphysema

**DOI:** 10.3389/fphys.2018.00121

**Published:** 2018-02-20

**Authors:** Mariana N. Machado, Flavia Mazzoli-Rocha, Natália V. Casquilho, Tatiana Maron-Gutierrez, Victor H. Ortenzi, Marcelo M. Morales, Rodrigo S. Fortunato, Walter A. Zin

**Affiliations:** ^1^Laboratory of Respiration Physiology, Carlos Chagas Filho Institute of Biophysics, Universidade Federal do Rio de Janeiro, Rio de Janeiro, Brazil; ^2^Laboratory of Immunopharmacology, Oswaldo Cruz Institute (FIOCRUZ), Rio de Janeiro, Brazil; ^3^Laboratory of Molecular Radiobiology, Carlos Chagas Filho Institute of Biophysics, Universidade Federal do Rio de Janeiro, Rio de Janeiro, Brazil; ^4^Laboratory of Cellular and Molecular Physiology, Carlos Chagas Filho Institute of Biophysics, Universidade Federal do Rio de Janeiro, Rio de Janeiro, Brazil

**Keywords:** pulmonary emphysema, papain, bone marrow-derived mononuclear cells, lung mechanics, histology, apoptosis, expression of dual oxidase

## Abstract

Murine papain-induced emphysema is a model that reproduces many of the features found in patients. Bone marrow-derived mononuclear cells (BMMC) have already been used to repair the alveolar epithelium in respiratory diseases, but not in the papain model. Thus, we hypothesized that BMMC could prevent the pathophysiological processes in papain-induced experimental emphysema. Female BALB/c mice received intratracheal instillation of 50 μL of saline (S groups) or papain (P groups, 10 IU/50 μl of saline) on days 1 and 7 of the experimental protocol. On the 14th day, 2 × 10^6^ BMMC of male BALB/c mice (SC21 and PC21) or saline (SS21 and PS21) were injected by the jugular vein. Analyses were done on days 14 (S14 and P14) and 21 (SS21, PS21, SC21, and PC21) of the protocol. qPCR evaluated the presence of the Y chromosome in the lungs of BMMC recipient animals. Functional residual capacity (FRC), alveolar diameter, cellularity, elastic fiber content, concentrations of TNF-α, IL-1β, IL-6, MIP-2, KC, IFN-γ, apoptosis, mRNA expression of the dual oxidase (DUOX1 and DUOX2), production of H_2_O_2_ and DUOX activity were evaluated in lung tissue. We did not detect the Y chromosome in recipients' lungs. FRC, alveolar diameter, polymorphonuclear cells (PMN) and levels of KC, MIP-2, and IFN-γ increased in P14 and PS21 groups; the changes in the latter were reverted by BMMC. TNF-α, IL-1β e IL-6 were similar in all groups. The amount of elastic fibers was smaller in P14 and PS21 than in other groups, and BMMC did not increase it in PC21 mice. PS21 animals showed increased DUOX activity and mRNA expression for DUOX1 and 2. Cell therapy reverted the activity of DUOX and mRNA expression of DUOX1. BMMC reduced mRNA expression of DUOX2. Apoptosis index was elevated in PS21 mice, which was reduced by cell therapy in PC21. Static compliance, viscoelastic component of elastance and pressure to overcome viscoelasticity were increased in P14 and PS21 groups. These changes and the high resistive pressure found on day 21 were reverted by BMMC. In conclusion, BMMC showed potent anti-inflammatory, antiapoptotic, antioxidant, and restorative roles in papain-triggered pulmonary emphysema.

## Introduction

The pathophysiology of pulmonary emphysema is complex (Gold-Global Initiative for Chronic Obstructive Lung Disease, 2017). The most accepted hypothesis regarding its development is the presence of an imbalance between the activity of proteases and anti-proteases in lung tissue, resulting in degradation of elastin (Janoff, 1985). Five main mechanisms account for the pathogenesis of pulmonary emphysema: (1) imbalance in the proteolytic/anti-proteolytic system; (2) alterations of the tissue injury/repair mechanism; (3) inflammation; (4) oxidative stress; and, (5) apoptosis of lung cells (Barnes, 2006, 2014; Yoshida and Tuder, 2007; Bagdonas et al., 2015; Gold-Global Initiative for Chronic Obstructive Lung Disease, 2017). Elastase elicits a chain of inflammatory response with involvement of alveolar macrophages and neutrophil influx into the pulmonary parenchyma that leads to an important release of proteases, overcoming the antiproteolytic defenses of the lower respiratory tract, often resulting in destruction of the pulmonary parenchyma (Gross et al., 1965; Roth, 2008; Laurell and Eriksson, 2013; Bagdonas et al., 2015).

The fibroproliferative response causing tissue remodeling begins almost immediately after the onset of the lung injury (Rocco et al., 2001; Bellingan, 2002). The accumulation of inflammatory cells and the entry of plasma into the alveolar spaces modify the alveolar microenvironment, leading to restoration of the alveolar architecture or to progressive fibrosis (Toews, 1999). Some mechanisms attempt to explain this “pulmonary maintenance/repair failure,” such as: increased cellular apoptosis (Imai et al., 2001; Majo et al., 2001; Tuder et al., 2003; Yokohori et al., 2004), reduction of cell proliferation and cellular chemotaxis (Carnevali et al., 1998; Rennard et al., 2006), among others.

Chronic inflammation in pulmonary emphysema is associated with an increase in different proinflammatory mediators, including TNF-α, IL-1β, IL-6, IL-12, IL-18 and chemokines such as IL-8, MIP-2, IFN-γ, MCP-1, MIP-1β, produced mainly by neutrophils and macrophages in the lung (Yoshida and Tuder, 2007; Churg et al., 2008; Roth, 2008; Bagdonas et al., 2015). These cytokines and proteinases eventually contribute to the development of emphysema by destroying components of the extracellular matrix (elastic fibers) (Calverley and Rennard, 2007; Barnes, 2014; Bagdonas et al., 2015).

The nicotinamide adenine dinucleotide phosphate (NADPH) oxidases, that represent one of the main sources of reactive oxygen species (ROS) in all biological systems, constitute a family of enzymes with seven isoforms: NOX1-5 and DUOX1-2 (Bedard et al., 2007; Kawahara et al., 2007; Sumimoto, 2008). NOX/DUOX enzymes are expressed along the respiratory tract on the surface of epithelial cells and fibroblasts (Bernard et al., 2014). The expression or inappropriate activation of NOX/DUOX in the lungs generate an excessive production of ROS, which includes hydrogen peroxide (H_2_O_2_), due to the imbalance of the antioxidant defense system (Lambeth, 2004). That imbalance is largely responsible for pulmonary tissue damage in various respiratory diseases, such as chronic inflammatory diseases (van der Vliet, 2008; Bernard et al., 2014).

The treatment of emphysema is still not well-defined, including surgery when there are bubbles large enough to compress the normal lung parenchyma and pleural cavity (Gulsen et al., 2017). In addition to lung transplantation, other therapies are used in emphysema: corticosteroids, bronchodilators, oxygen therapy, and pulmonary rehabilitation, but none of them seems to prevent the progression of the disease and reduce mortality, albeit they can improve patients' quality of life (Gold-Global Initiative for Chronic Obstructive Lung Disease, 2017). Therefore, cell therapy techniques open a new and promising perspective for the treatment of pulmonary emphysema.

The ability of stem cells to release different mediators that influence lung processes like inflammation and remodeling stems from their paracrine action (Conese et al., 2013; de Oliveira et al., 2017). Such mechanism was first identified by observing that the systemic administration of bone marrow-derived stem cells inhibited the increase in the expression of various proinflammatory and profibrogenic cytokines in models of acute lung injury and pulmonary fibrosis (Ortiz et al., 2003, 2007; Conese et al., 2013; de Oliveira et al., 2017).

Currently, despite advances in the understanding of the pathophysiology of emphysema and the introduction of new therapeutic interventions, control of this condition is still lacking. Therefore, since stem-cell therapy has been shown to have anti-inflammatory and antifibrogenic activity in several lung diseases, such as asthma (Abreu et al., 2011; Conese et al., 2013), COPD (Huh et al., 2011; Cruz et al., 2012; Antunes et al., 2014; Jin et al., 2015), and LPS-triggered lung inflammation (Yamada et al., 2004; Gupta et al., 2007; Xu et al., 2007). Kim et al. (2014) tested mesenchymal stem cells in an elastase-induced model of emphysema to study the timeline and concentration of these cells. In this context, we hypothesized that stem cells might present beneficial effects on pulmonary emphysema and, hence, aimed to test whether bone marrow-derived mononuclear cells (BMMC) could treat a murine model of papain-induced pulmonary emphysema.

## Methods

### Animal preparation

The animals were sedated by inhalation of sevoflurane, weighed (model BR, Filizola Industries SA, SP, Brazil), and saline or papain was injected into the trachea using an insulin syringe. This procedure last about 3 min.

Fifteen male BALB/c mice (20–25 g) were quickly euthanized by cervical dislocation. BMMC were aspirated from their femur and tibia by flushing the bone marrow cavity with Dulbecco's modified Eagle's medium (DMEM, Life Technologies, Grand Island, NY, USA). After a homogeneous cell suspension was obtained, the cells were centrifuged (4,000 g for 10 min), re-suspended in DMEM and added to Ficoll-Hypaque (Histopaque 1083; Sigma-Aldrich, St. Louis, MO, USA), centrifuged again (5,000 g, 30 min) and supplemented with sterile PBS. Cells were counted in a Neubauer chamber with Trypan Blue for evaluation of viability. Cell characterization was performed by flow cytometry using specific antibodies (Conget and Minguell, 1999; Maron-Gutierrez et al., 2011).

**Figure 1:**
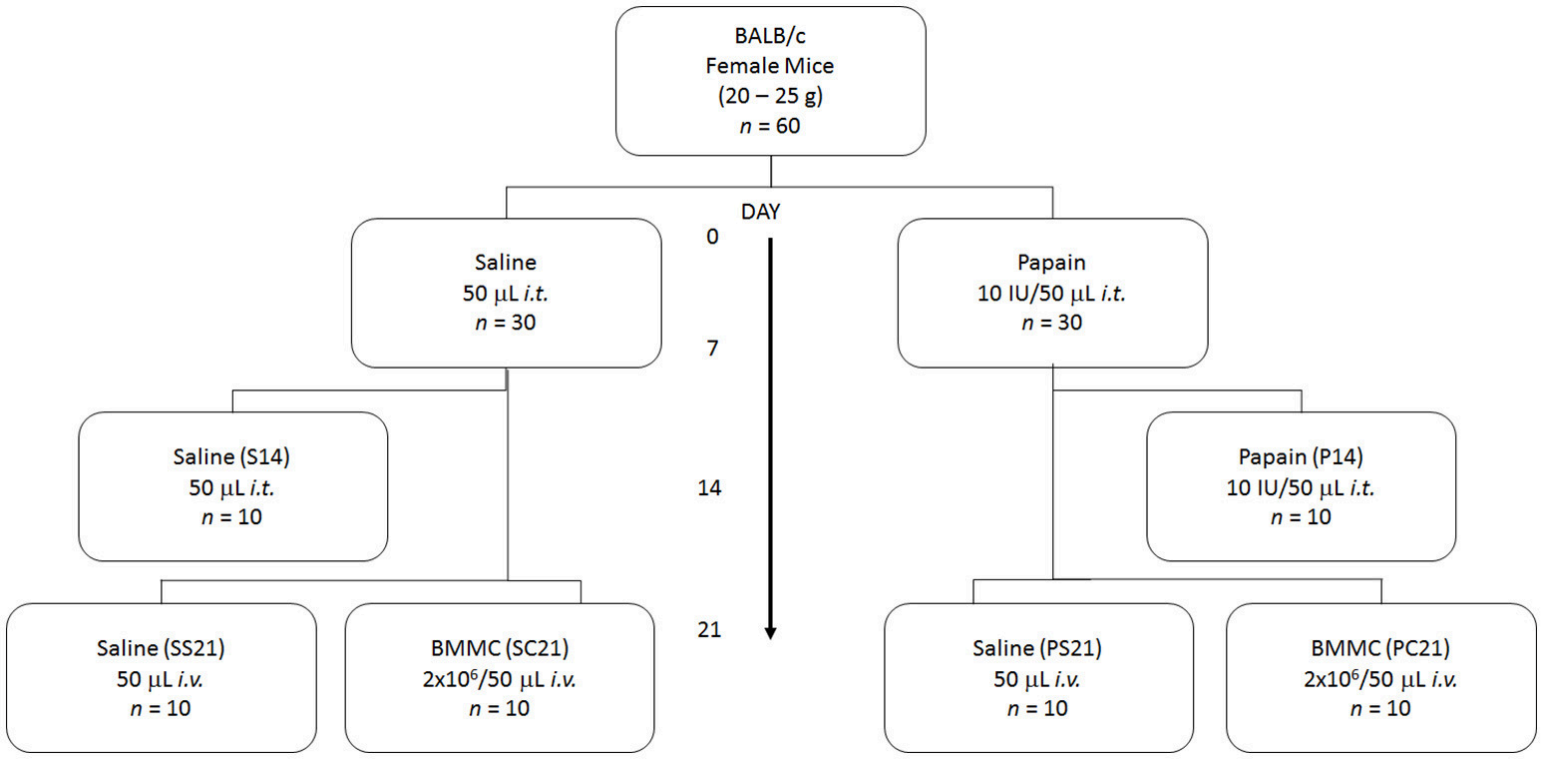
Schematic flow chart and timeline of study design. On days 0 and 7 all mice received intratracheal instillation (*i.t*.) of saline solution or papain. On day 14 all received intravenous injection (*i.v*.) of saline or BMMC. Data were analyzed on days 14 and 21. S, intratracheal instillation of 50 μL of saline; P, intratracheal instillation of 10 IU of papain; S, injection of 50 μL of saline; C, injection of bone marrow mononuclear cell (2 × 10^6^/50 μL of saline).

Figure 1 shows that 60 female BALB/c mice (20–25 g) were randomly divided into six groups. In S14 (*n* = 10), SS21 (*n* = 10), and SC21 (*n* = 10) groups, mice were intratracheally (*i.t*.) injected with 50 μL of sterile saline solution (0.9% NaCl) on days 1 and 7 of the experimental protocol. In P14 (*n* = 10), PS21 (*n* = 10), and PC21 (*n* = 10) groups, mice were i.t. injected with 50 μL of sterile saline solution (0.9% NaCl) containing 10 IU of papain (0.2 IU/μL) on days 0 and 7 of the experimental protocol. Papain (USP 225310, Becton Dickinson, Franklin Lakes, NJ, USA) had been previously activated in 0.1 M sodium phosphate buffer containing 10 mM EDTA, 0.4 NaCl and 5 mM dithiothreitol for 10 min at 40°C (Machado et al., 2014). On the 14th day, 2 × 10^6^ BMMC from male BALB/c mice suspended in 50 μL of sterile saline solution (SC21 and PC21 groups) or 50 μL of sterile saline solution (0.9% NaCl) (SS21 and PS21 groups) were injected into the jugular vein. Histopathological parameters and pulmonary mechanics were analyzed on the 14th (S14 and P14 groups) and 21st (SS21, PS21, SC21, and PC21 groups) days of the protocol.

All animals received humane care in compliance with the “Principles of Laboratory Animal Care” formulated by the National Society for Medical Research,the “Guide for the Care and Use of Laboratory Animals” prepared by the National Academy of Sciences, USA, the “APS's Guiding Principles in the Care and Use of Vertebrate Animals in Research and Training,” and the National Council for Controlling Animal Experimentation, Ministry of Science, Technology and Innovation (CONCEA/MCTI), Brazil. The experiments were approved by the Ethics Committee on the Use of Animals, Health Sciences Center, Federal University of Rio de Janeiro (Protocol IBCCF 130/14).

### Pulmonary mechanics

On the 14th and 21st days after the first instillation, the animals were sedated with diazepam (1 mg *i.p*.) and anesthetized with pentobarbital sodium (20 mg/kg body weight *i.p*.), paralyzed with pancuronium bromide (0.1 mg/kg body weight *i.v*.), and mechanically ventilated in air (Samay VR15, Universidad de la Republica, Montevideo, Uruguay) with a frequency of 100 breaths/min, tidal volume of 0.2 mL, inspiratory flow of 1 mL/s, and positive end-expiratory pressure (PEEP) of 2 cmH_2_O (Saldiva et al., 1992). The anterior chest wall was surgically removed.

Lung mechanics was determined as previously described (Machado et al., 2014). Briefly, we determined lung resistive (ΔP1) and viscoelastic/inhomogeneous (ΔP2) pressures, static elastance (Est), and viscoelastic component of elastance (ΔE) by the end-inflation occlusion method (Bates et al., 1985). ΔP1 selectively reflects airway resistance, and ΔP2 represents stress relaxation or viscoelastic properties and mechanical heterogeneities of the lung (Bates et al., 1988; Saldiva et al., 1992). Lung mechanics were measured 10–15 times in each animal.

### Histological study

Heparin (1000 IU) was injected into the abdominal vena cava right after the determination of respiratory mechanics. The trachea was clamped at end-expiration, and the abdominal aorta and vena cava were sectioned, yielding a massive hemorrhage that quickly euthanized the animals. The lungs were removed *en bloc*.

Functional residual capacity (FRC) was determined by the volume displacement technique (Scherle, 1970).

The right lung was frozen in liquid N_2_ for posterior biochemical analyses, whereas the left lung was submerged in buffered 10 % formaldehyde (Millonig's phosphate buffer: 100 mL HCHO, 900 mL H_2_O, 18.6 g NaH_2_PO_4_, 4.2 g NaOH). After fixation, the tissue was embedded in paraffin. Four-μm-thick slices were cut and stained with hematoxylin-eosin for the determination of lung cellularity and alveolar diameter or orcein for the identification of elastic fibers.

Lung slides stained with H-E were analyzed by optical microscopy (Axioplan, Zeiss, Oberkochen, Germany). Quantitative analysis was performed by the point-counting technique with a coherent system made of a 100-point and 50-line (1,250-μm-long each) grid (Weibel, 1990) coupled to a conventional light microscope across 10 random non-overlapping microscopic fields (Axioplan, Zeiss, Oberkochen, Germany). The amount of polymorphonuclear (PMN) cells in the pulmonary tissue was evaluated at 1,000x magnification by counting points falling on PMN cells and dividing it by the total number of points falling on overall tissue area in each microscopic field (Gundersen et al., 1988; Weibel, 1990). For determination of mean alveolar diameter (Lm) the number of alveolar intercepts in 20 random fields in each sample was determined at 200x magnification, and Lm was calculated as the sum of line segments (1,250 μM each)/number of intercepts.

**Figure 2:**
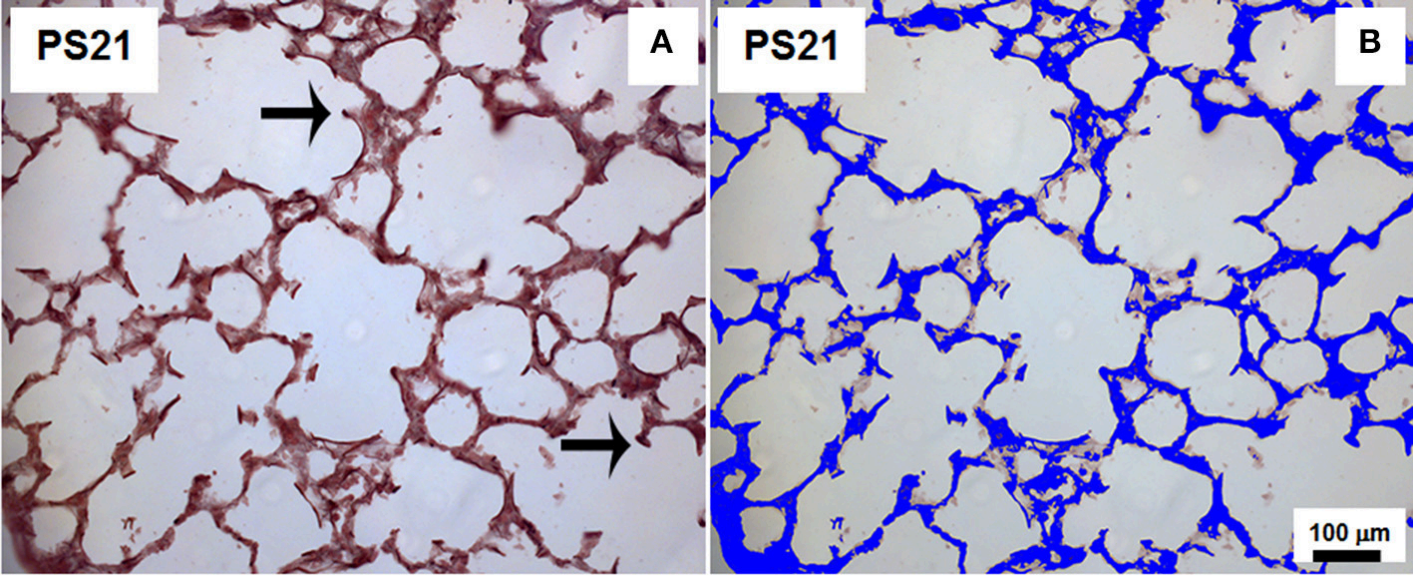
Method of analysis of the area occupied by elastic fibers. **(A)** represents an orcein-stained slide depicting elastic fibers as dark brown lines (arrows) and **(B)** illustrates the staining of total lung tissue area in blue. This representative animal belonged to PS21 group that received papain. 400x magnification.

The area of lung parenchyma occupied by elastic fibers (Weigert's resorcin fuchsin method with oxidation) (Fullmer et al., 1974) was identified in a blinded manner by the point-counting technique on images captured across 10 random non-coincident fields (400x magnification). The quantification was done on captured high quality images (2,048 × 1,536 pixels) using the Image Pro Plus 4.5.1 software (Media Cybernetics, Silver Spring, MD, USA) (Figure 2). Results were expressed as percentage of points falling on elastic fibers divided by the number of points hitting lung tissue.

### Detection of Y chromosome DNA

Quantification of murine Y chromosome in lung tissue was achieved by quantitative real-time PCR. Five nanograms of DNA were used in a real-time PCR reaction with the SYBR Green detection kit run in a 7000 Sequence Detection System thermocycler according to the manufacturer's instructions (Applied Biosystems, Foster City, CA, USA). The presence of the Y chromosome in the samples was calculated by comparing the value of crosspoint threshold of amplification with the standard curve.

The following PCR primers were used to evaluated SRY (Sex determining region on Y): 5-TCATCGGAGGGCTAAAGTG-3 (forward); 5-CAACCTTCTGCAGTGGGAC-3 (reverse) and GAPDH (Glyceraldehyde 3-phosphate dehydrogenase): 5-CCACCAACTGCTTAGCCC-3 (forward); 5-GACACCTACAAAGAAGGGT CCA-3 (reverse).

### Analysis of cytokines

Samples of lung cytosol were homogenized and analyzed by ELISA for the detection of the inflammatory cytokines: tumor necrosis factor-alpha (TNF-α), interleukin 1β (IL-1β), interleukin 6 (IL-6), keratinocyte derived chemokine (KC), macrophage inflammatory protein-2 (MIP-2), and interferon gamma (IFN-γ) using high sensitivity kits (R&D Systems Inc., Minneapolis, MN, USA). The total protein content in lung homogenate samples was determined by Bradford's method (1976). Final values are expressed in pg/mg protein.

### Apoptosis index

Apoptotic cells in lung tissue were detected by terminal deoxynucleotidyl transferase biotin-dUTP nick end labeling (TUNEL) assay, according to the manufacturer's instructions (S7100, EMD Millipore, Billerica, MA, USA). Twenty fields displaying alveolar septa were randomly studied. TUNEL positive cells (400x) in the lung were analyzed using the software QCapture Pro 7 Image and Analysis Software (QImaging, Surrey, BC, Canada). Apoptosis index in lung tissue is the percentage ratio between the number of TUNEL positive cells (apoptosis) and the total number of cells in each field.

### Real-time reverse transcription-polymerase chain analysis

Total RNA was extracted from the mice lung using the RNeasy® Mini Kit (Qiagen, Hilden, Germany), following the manufacturer's instructions. After DNAse treatment, reverse transcription was followed by real-time polymerase chain reaction (PCR), as previously described (Schmittgen and Livak, 2008). Expression of the target genes was normalized to a control gene [acidic ribosomal phosphoprotein P0 (36β4)], and the relative fold changes were calculated using the ^ΔΔ^CT method. The specific oligonucleotides were used: Oxidase dual (DUOX) 1, forward: 5-TCCTATGTTCCTGTACCTTTGTG-3, reverse: 5-GTCCCACCTCCATCTTGAATC-3; Oxidase dual (DUOX) 2, forward: 5-CTCTACTGGATGACTGGAAACC-3, reverse: 5-AGTCAGGTCTGTTTTCTTGCC-3; 36β4, forward: 5-CAACCCAGCTCTGGAGAAAC-3, reverse: 5-GTTCTGAGCTGGCACAGTGA-3.

### Measurement of H_2_O_2_ generation and duox activity

Extracellular H_2_O_2_ generation was quantified by the Amplex Red/HRP assay (Molecular Probes, Eugene, OR, USA) that detects the accumulation of a fluorescent oxidized product. H_2_O_2_ release was quantified (nmol H_2_O_2_/h per mg protein) using standard calibration curves. To determine the specific activity of DUOX, the activity of H_2_O_2_ obtained in the presence of calcium was subtracted from the activity in the absence of calcium (Fortunato et al., 2010; Mühlbauer et al., 2010).

### Statistical analysis

SigmaStat 11 statistical package (Systat Software, San Jose, CA, USA) was used. The normality of the data (Kolmogorov-Smirnov test with Lilliefors' correction) and the homogeneity of variances (Levene median test) were tested. Since in all instances both conditions were satisfied two-way ANOVA was performed followed by Tukey *post-hoc* test when necessary (*p* < 0.05).

## Results

Pulmonary mechanics was measured in groups S14, P14, SS21, PS21, SC21, and PC21 (Tables 1, 2). Lung static elastance (Est) and viscoelastic component of elastance (ΔE) were higher in P14 than in S14 group. Both parameters reflect an increase in pulmonary stiffness. Exposure to papain also augmented ΔP2 in P14 mice, while ΔP1 was similar in both groups (Table 1). On the 21st day of the experimental protocol, pulmonary mechanics was determined in the other experimental groups. The animals that underwent BMMC therapy (PC21) showed functional parameters statistically similar to SS21 (Table 2).

**Table 1:** Mechanics, histology, and inflammation markers in lung parenchyma 14 days after exposure.

	**S14**	**P14**
**MECHANICS**		
ΔP1 (cmH_2_O)	0.66 ± 0.04	0.79 ± 0.05
ΔP2 (cmH_2_O)	0.87 ± 0.05	1.16 ± 0.08^*^
Est (cmH_2_O/mL)	26.43 ± 0.80	34.58 ± 2.01^*^
ΔE (cmH_2_O/mL)	4.45 ± 0.28	5.79 ± 0.34^*^
**HISTOLOGY**		
PMN (cells × 10^−3^/μm^2^)	1.17 ± 0.01	1.62 ± 0.16^*^
Lm (μm)	30.67 ± 0.54	34.79 ± 0.56^*^
FRC (mL)	0.19 ± 0.01	0.22 ± 0.01^*^
Elastic fiber (% tissue area)	25.77 ± 1.86	19.77 ± 2.12^*^
**CYTOKINES**		
TNF-α (pg/mg ptn)	87.30 ± 3.02	92.84 ± 7.99
IL-1β (pg/mg ptn)	139.58 ± 8.66	140.34 ± 7.44
IL-6 (pg/mg ptn)	156.32 ± 15.60	178.56 ± 17.09
KC (pg/mg ptn)	94.06 ± 5.64	112.52 ± 4.81^*^
MIP-2 (pg/mg ptn)	13.84 ± 0.53	22.78 ± 2.39^*^
IFN-γ (pg/mg ptn)	118.05 ± 13.40	196.02 ± 8.68^*^

*Values are mean ± SD of 5–10 animals/group. Female BALB/c mice received an intratracheal instillation of either 50 μL of sterile saline (0.9% NaCl, S14) or 10 IU of papain (0.2 IU/μL of saline, P14) on days 0 and 7 and were analyzed on day 14. ΔP1 and ΔP2, resistive and viscoelastic/inhomogeneous pressures, respectively; Est, static elastance; ΔE, viscoelastic component of elastance; PMN, polymorphonuclear cells; Lm, mean linear intercept; FRC, functional residual capacity; TNF-α, tumor necrosis factor alfa; IL-1β, interleukin 1-beta; IL-6, interleukin 6; KC, keratinocyte derived chemokine; MIP-2, macrophage inflammatory protein-2; IFN-γ, interferon gamma; ptn, protein*.

* *Significantly different from S14 group (p < 0.05)*.

**Table 2:** Mechanics, histology, and inflammation markers in lung parenchyma 21 days after exposure.

	**SS21**	**PS21**	**SC21**	**PC21**
**MECHANICS**				
ΔP1 (cmH_2_O)	0.39 ± 0.05	0.76 ± 0.03^*^	0.60 ± 0.03	0.69 ± 0.03
ΔP2 (cmH_2_O)	1.02 ± 0.06	1.30 ± 1.12^*^	1.01 ± 0.06	0.92 ± 0.03
Est (cmH_2_O/mL)	20.68 ± 1.11	30.85 ± 1.32^*^	22.14 ± 1.06	25.93 ± 0.98
ΔE (cmH_2_O/mL)	5.14 ± 0.28	6.60 ± 0.59^*^	5.14 ± 0.32	4.73 ± 0.16
**HISTOLOGY**				
PMN (cells × 10^−3^/μm^2^)	1.02 ± 0.05	2.99 ± 0.17^*^	1.19 ± 0.03	1.15 ± 0.09
Lm (μm)	30.50 ± 0.24	34.66 ± 0.32^*^	29.56 ± 0.19	29.13 ± 0.07
FRC (mL)	0.19 ± 0.01	0.24 ± 0.01^*^	0.18 ± 0.01	0.20 ± 0.02
Elastic fiber (% tissue area)	24.35 ± 3.52	4.58 ± 1.11^*^	21.70 ± 3.05	4.55 ± 0.76^*^, ^**^
**CYTOKINES**				
TNF-α (pg/mg ptn)	70.73 ± 8.66	93.56 ± 3.70	96.83 ± 7.23	85.90 ± 11.05
IL-1β (pg/mg ptn)	126.62 ± 6.35	130.18 ± 14.92	127.36 ± 7.14	127.54 ± 21.65
IL-6 (pg/mg ptn)	132.99 ± 20.32	120.18 ± 11.08	130.85 ± 9.49	103.18 ± 19.46
KC (pg/mg ptn)	113.03 ± 10.16	163.18 ± 11.2^*^	115.18 ± 17.18	90.15 ± 5.84^#^
MIP-2 (pg/mg ptn)	11.69 ± 0.49	22.15 ± 2.66^*^	15.17 ± 2.19	17.97 ± 2.88
IFN-γ (pg/mg ptn)	111.31 ± 17.66	151.24 ± 10.94^*^	122.89 ± 7.85	103.18 ± 19.46

*Values are mean ± SD of 5–10 animals/group. Female BALB/c mice received an intratracheal instillation of either 50 μL of sterile saline (0.9% NaCl, S) or 10 IU of papain (0.2 IU/μL of saline, P) on days 0 and 7. Bone marrow-derived mononuclear cells (BMMC) came from male donor mice. On day 14 mice were intravenously injected with 2 × 10^6^ BMMC (SC21 and PC21) or with saline solution (SS21 and PS21). Mice were analyzed on day 21. ΔP1 and ΔP2, resistive and viscoelastic/inhomogeneous pressures, respectively; Est, static elastance; ΔE, viscoelastic component of elastance; PMN, polymorphonuclear cells; Lm, mean linear intercept; FRC, functional residual capacity; TNF-α, tumor necrosis factor alfa; IL-1β, interleukin 1-beta; IL-6, interleukin 6; KC, keratinocyte derived chemokine; MIP-2, macrophage inflammatory protein-2; IFN-γ, interferon gamma; ptn, protein*.

* Represent statistically significant differences (p < 0.05) in relation to their respective controls (SS21 and SC21);

** Similar to PS21 animals;

# *Statistically significant differences (p < 0.05) from the other groups*.

BMMC were able to revert the increase in FRC and Lm triggered by the papain-induced pulmonary emphysema model (Tables 1, 2). Pulmonary hyperinflation was already established in the lung when the animals received BMMC. Table 2 shows that exposure to papain caused an increase in FRC, which was reverted by cell therapy.

**Figure 3:**
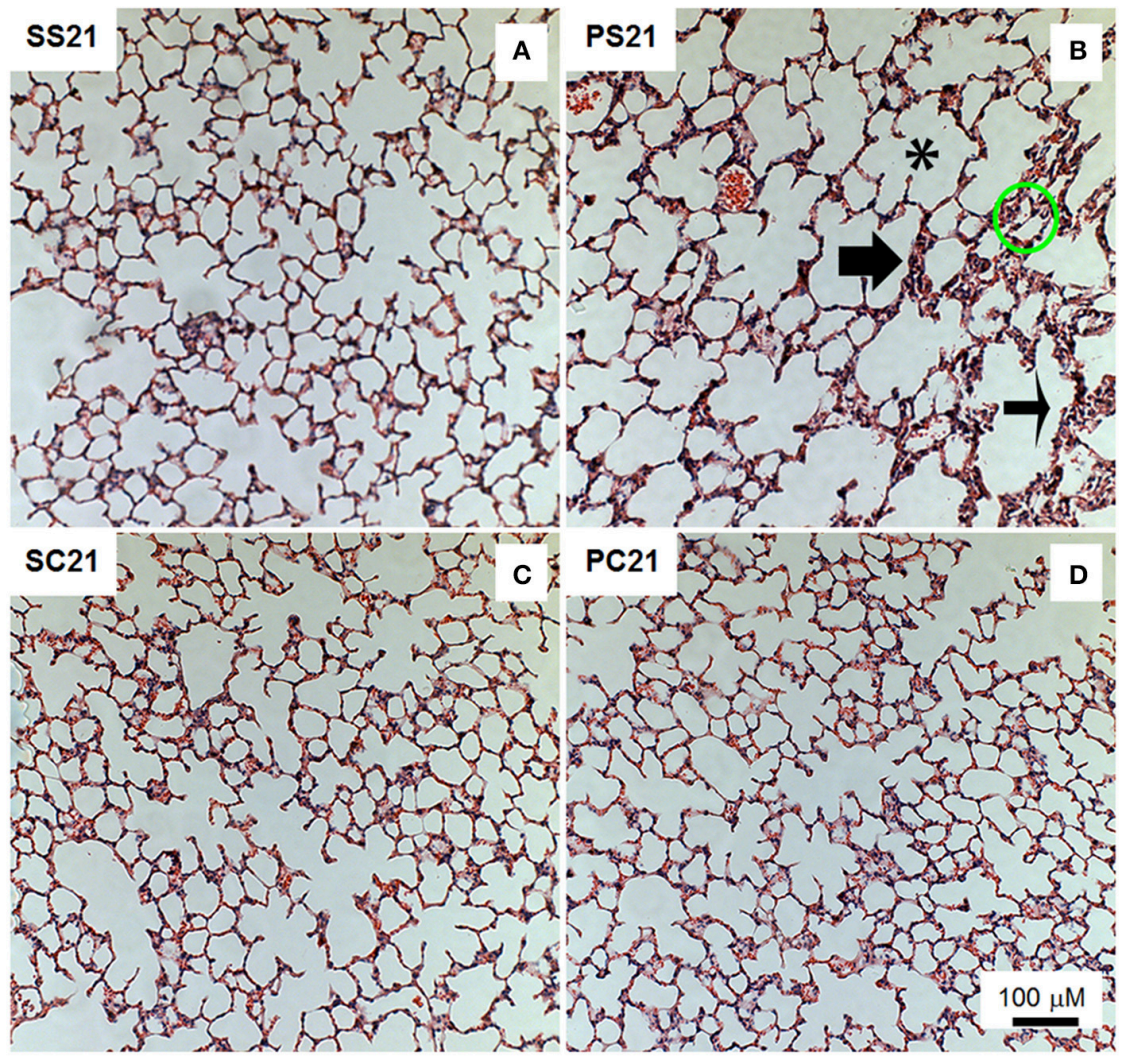
Photomicrographs of lung parenchyma stained with hematoxylin-eosin. Measurements were done 21 days after the exposure of animals to papain. Female BALB/c mice received an intratracheal instillation of either 50 μL of sterile saline 0.9% NaCl (S) or 10 IU of papain (0.2 IU/μL in 50 μL of saline) (P) on days 0 and 7. On day 14 mice were intravenously injected with 2 × 10^6^ BMMC (SC21 and PC21) or with saline solution (SS21 and PS21). Mice were studied on day 21. ^*^Alveolar rupture; thin arrow: thickened septa; thick arrow: cellular infiltrate; and circles: alveolar collapse.

There was a significant reduction in elastic fiber deposition in P14 group (Table 1) and PS21 groups in relation to their respective controls (S14 and SS21 groups, respectively) (Table 2). However, in the mice exposed to papain and treated with BMMC (PC21 group) elastic fiber deposition remained similar to PS21 group. The number of polymorphonuclear cells (PMN) increased in the alveoli and alveolar septa in the PS21 group in relation to the other animals. In BMMC-treated mice (PC21), less PMN cells were observed in relation to the papain group (PS21) (Table 2 and Figure 3). Note that PMN cells infiltration was already present in lung tissue at the time the mice received cell therapy (P14, 14th day of the experimental protocol) (Table 1).

Higher levels of KC, IFN-γ, and MIP-2 were found in PS21 mice than in the other groups studied on the 21st day of the protocol. Treatment with BMMC reverted the increase in cytokine levels (PC21 group) (Table 2). The levels of KC, IFN-γ, and MIP-2 in lung tissue were already augmented when the mice received cell therapy (P14 group) (Table 1).

**Figure 4:**
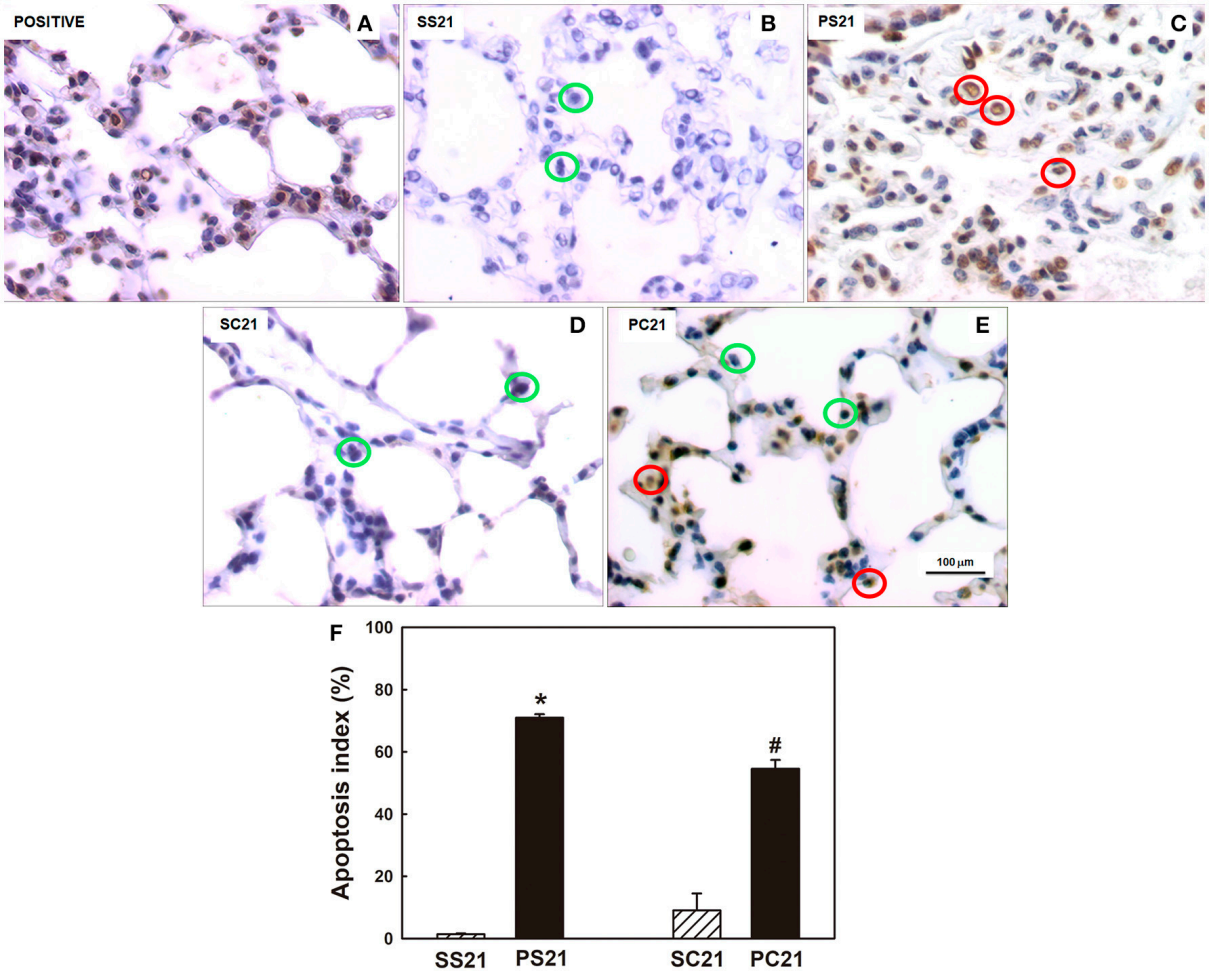
Determination of apoptosis in lung tissue. **(A–E)** Photomicrographs (400x) of the pulmonary parenchyma depicting *in situ* cells apoptosis, as revealed by brownish nuclei. **(F)** Quantification of the apoptosis index was determined by TUNEL positive cells/total cells. Female BALB/c mice received an intratracheal instillation of either 50 μL of sterile saline 0.9% NaCl (S) or 10 IU of papain (0.2 IU/μL in 50 μL of saline) (P) on days 0 and 7. On day 14 mice were intravenously injected with 2 × 10^6^ BMMC (SC21 and PC21) or with saline solution (SS21 and PS21). Mice were studied on day 21. Green circles indicate normal cells and red circles indicate apoptotic cells. Values are mean + *SD* of 10 animals/group. ^*^Significantly different from control (SS21); ^#^Significantly different from control (SC21) and PS21 (*p* < 0.05).

PS21 group presented a higher number of apoptotic cells in the lung tissue than the other groups studied on the 21st day. The animals that received papain and subsequently BMMC therapy had reduced numbers of apoptotic cells compared to the groups that were not treated after papain exposure (Figures 4A–E). A higher apoptosis index was observed in the PS21 group in relation to the other groups studied on the 21st day. Treatment with BMMC reduced (PC21 group), but did not reverted the degree of apoptosis (Figure 4F).

**Figure 5:**
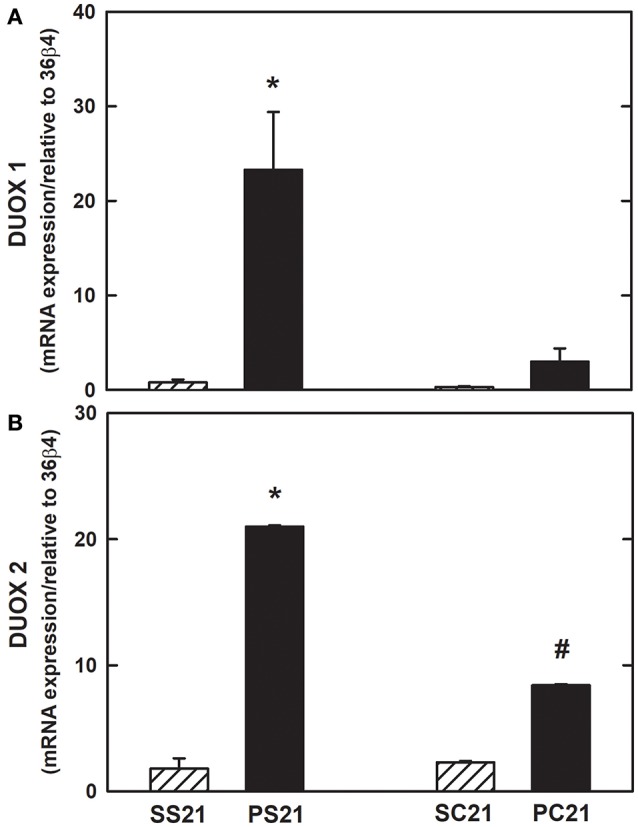
Expression of mRNA for oxidase dual (DUOX) in lung tissue. mRNA expression of NADPH oxidases in the lung by real-time polymerase chain reaction (PCR). **(A)** DUOX1 and **(B)** DUOX2. Female BALB/c mice received an intratracheal instillation of either 50 μL of sterile saline 0.9% NaCl (S) or 10 IU of papain (0.2 IU/μL in 50 μL of saline) (P) on days 0 and 7. On day 14 mice were intravenously injected with 2 × 10^6^ BMMC (SC21 and PC21) or with saline solution (SS21 and PS21). Mice were studied on day 21. Values are mean + *SD* of 10 animals/group. ^*^Significantly different from control (SS21); ^#^Significantly different from control (SC21) and PS21 (*p* < 0.05).

Expression of mRNA for DUOX1 and DUOX2 increased in lung tissue of PS21 group when compared to the other groups (Figures 5A,B, respectively). The administration of 2 × 10^6^ BMMC could attenuate the expression of DUOX1 and DUOX2 mRNAs.

**Figure 6:**
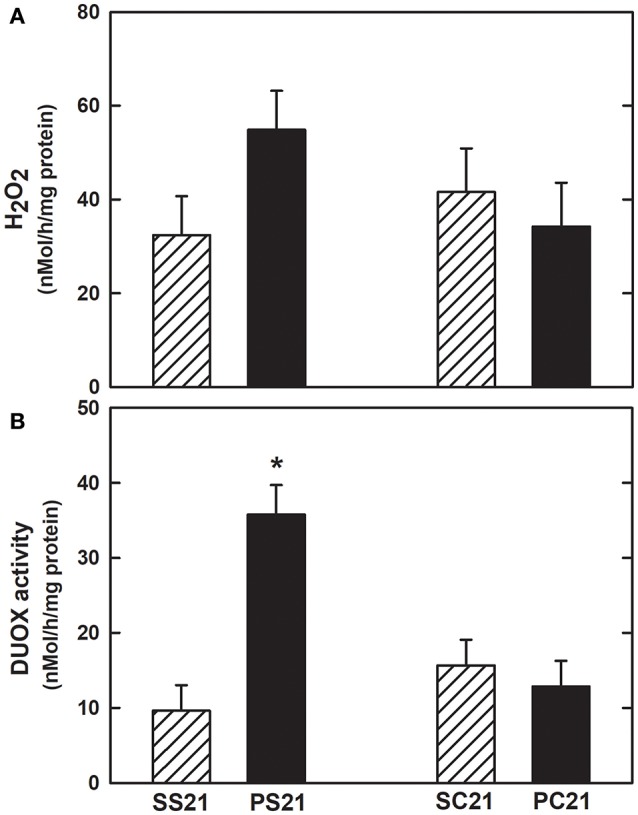
Hydrogen peroxide (H_2_O_2_) production and oxidase dual (DUOX) activity in lung tissue**. (A)** H_2_O_2_ generation and **(B)** DUOX activity. Female BALB/c mice received an intratracheal instillation of either 50 μL of sterile saline 0.9% NaCl (S) or 10 IU of papain (0.2 IU/μL in 50 μL of saline) (P) on days 0 and 7. On day 14 mice were intravenously injected with 2 × 10^6^ BMMC (SC21 and PC21) or with saline solution (SS21 and PS21). Mice were studied on day 21. Values are mean + *SD* of 10 animals/group. ^*^Significantly different from other groups (*p* < 0.05).

Figures 6A,B show the total H_2_O_2_ generation and DUOX activity in the presence of calcium. Increased H_2_O_2_ generation and calcium-stimulated DUOX activity were observed in lung tissue of PS21 group in relation to the other groups tested on the 21st day, reinforcing the hypothesis that therapy with 2 × 10^6^ BMMC may have an antioxidant effect.

No specific signal of the male target gene was detected in the SS21 (0.00063 ± 0.00042), SC21 (0.00091 ± 0.00105), and PC21 (0.00045 ± 0.00063) groups.

## Discussion

The present study showed that the intravenous use of BMMCs in an experimental model of papain-induced pulmonary emphysema resulted in a plethora of morpho-functional improvements in the pulmonary parenchyma. BMMC infusion was able to improve elastic, viscoelastic and resistive pulmonary mechanics, revert histological changes, revert inflammatory markers, decrease the rate of apoptosis, and, in a pioneer way, reduce the oxidative stress, via enzymes DUOX1 and DUOX2 in papain-treated mice. BMMC did not stimulate the deposition of elastic fibers. All these outcomes might be attributed to the BMMC cells because there was no male donor mice cells (Y chromosome) in the lung tissue of the female recipient mice.

Previous works (Marco et al., 1972; Fló et al., 1985; Fusco et al., 2002; Borzone et al., 2007; Lopes et al., 2009) used a model of pulmonary emphysema that showed several morphofunctional changes after intratracheal instillation of papain. Our use of a papain-induced pulmonary emphysema model was based on the study by Machado et al. (2014), who observed inflammatory, oxidative and structural alterations consistent with those observed in human emphysema 21 days after the intratracheal instillation of papain. Those alterations included increased airspace volume measured by functional residual capacity (FRC) and mean alveolar diameter, influx of polymorphonuclear cells in the pulmonary parenchyma, reduction in the amount of elastic and collagen fibers by area of lung tissue and increase in resistive and pulmonary elastic properties.

The beneficial effect of cell therapy on lung function is well-documented (Lassance et al., 2009; Huh et al., 2011; Maron-Gutierrez et al., 2011; Schweitzer et al., 2011; Longhini-Dos-Santos et al., 2013). Maron-Gutierrez et al. (2011) observed that cell therapy (2 × 10^6^ cells/mice) reverted all mechanical alterations in a pulmonary silicosis model. BMMC act in the remodeling and inflammatory process probably because of their paracrine effect (Takahashi et al., 2003; Ortiz et al., 2007; Katsha et al., 2011; Conese et al., 2013). Corroborating these findings, BMMC (2 × 10^6^ cells/mice) reverted the changes in the elastic and resistive pulmonary components triggered by the instillation of papain, possibly by a paracrine action, since we did not find the cells of the donor animals (Y chromosome) in the lung tissue of female mice treated with BMMC.

Cell therapy is effective to regenerate destroyed alveolar structures (Huh et al., 2011; Schweitzer et al., 2011; Longhini-Dos-Santos et al., 2013). Huh et al. (2011) reported, in a model of lung emphysema induced by cigarette smoke, that BMMC (6 × 10^6^ cells/animal) can repair damaged alveoli. Adipose tissue-derived cell therapy (3 × 10^5^ cells/animals) repaired lung tissue after 3 or 4 months of exposure to cigarette smoke (Schweitzer et al., 2011). In murine models of elastase-induced emphysema Longhini-Dos-Santos et al. (2013) and Cruz et al. (2012) used 2.8 × 10^6^ and 2.0 × 10^6^ BMMC, respectively, to repair destroyed lung structures. Kim et al. (2014) reported the optimal therapeutic dose of bone marrow derived mesenchymal cells (5 × 10^4^) in an animal model of emphysema/COPD. The administration of 2 × 10^6^ BMMC acts in a prophylactic or therapeutic manner, improving lung function by reducing inflammation (Maron-Gutierrez et al., 2011) and fibrosis (Lopes-Pacheco et al., 2014). Thus, we used BMMC in a therapeutic approach, administering them after the lesion was established (P14 group). It should be stressed that in the present study we opted for cell therapy with the mononuclear fraction of cells derived from bone marrow (2 × 10^6^ cells/animal), since that would require less manipulation, favoring a possible future clinical use in humans.

In the present study, FRC and mean alveolar diameter augmented, and the total amount of elastic fibers in the lung parenchyma fell (P14 and PS21 animals), demonstrating the destruction of the pulmonary structure and the disarrangement of extracellular matrix fibers, central features of emphysema (Johanson et al., 1973). It should be stressed that these changes were already established before BMMC therapy (P14 group). BMMC reverted the increased FRC, partially reduced mean alveolar diameter, but not increased the amount of elastic fibers per lung tissue area (Table 2). Pastor et al. (2006) found intense intra-alveolar exudation at 12 h after intratracheal instillation of papain, dilated alveolar ducts at 3 days after exposure, and several fenestrations in the alveolar wall at 60 days. In a model of pulmonary emphysema induced by elastase, Cruz et al. (2012) demonstrated that BMMC administration preserves the amount of elastic fibers, reduces pulmonary hyperinflation, infiltration of inflammatory cells and deposition of collagen fibers in the lung parenchyma. Consistent with these findings, BMMC therapy presented a reparative effect in our study.

Most of the reports about cell therapy describing increased elastic fiber content in the lung used porcine elastase to develop a model of emphysema (Cruz et al., 2012; Antunes et al., 2014). We used papain, instead. In this model other authors report that 1 week after instillation of papain the elastin content initially decreases (Mahadeva and Shapiro, 2002), yet the expression of messenger RNA for elastin and deposition of new elastic fibers, although disorganized, has been observed (Shapiro, 2000). Lucey et al. (1998) demonstrated increased elastin mRNA expression in the pleura, blood vessels, and airways in a murine model of elastase-induced emphysema. Within the alveoli, elastin was observed mainly in the alveolar septa (Lucey et al., 1998; Shapiro, 2000). After 3 weeks of intratracheal instillation of the protease, there is intense destruction of the pulmonary parenchyma (alveoli and capillaries) and abnormal repair of the elastic fibers, contributing to pulmonary hyperinflation (Johanson et al., 1973; Kuhn et al., 1976), followed by accumulation of neutrophils and macrophages in the lung (Shapiro, 2000; Mahadeva and Shapiro, 2002). The alveolar destruction remains intense during the first month after the instillation of papain, and then stabilizes (Johanson et al., 1971; Shapiro, 2000; Pastor et al., 2006). In this line, we found that at 21 days after instillation papain was still contributing to disrupt elastic fibers (Tables 1, 2). Not surprisingly, BMMC could not revert the change of papain-induced elastic fiber content in lung tissue.

In a model of pulmonary elastase-induced emphysema, Cruz et al. (2012) reported that an intravenous BMMC therapy administered 3 h after the first instillation of elastase reduced alveolar collapse, hyperinflation, number of mononuclear cells, neutrophils, and collagen fiber deposition in lung tissue. Airway epithelium and alveolar-capillary membrane damage and less elastic fiber breakdown, and, the degree of lung apoptotic cells and caspase-3 expression were diminished. On the other hand, this model of early administration of BMMC increased elastic fibers in the alveolar septa. Thus, the aggressor is different from ours, as well as the protocol, now allowing a direct comparison between their results and ours.

An increased number of apoptotic cells (TUNEL positive) was observed in lung tissues of papain-treated animals that was reduced by BMMC therapy (Figure 4). In a murine model of papain-induced pulmonary emphysema, mesenchymal cell therapy reduced apoptosis of lung tissue cells (Zhen et al., 2010), which is consistent with our findings. Apoptosis plays a critical role in maintaining homeostasis of normal tissue and is in balance with cell proliferation and differentiation. Programmed cell death allows the elimination of unwanted, damaged, or infected cells (Demedts et al., 2006). There is increasing evidence that disturbance of the balance between apoptosis and proliferation in lung tissue contributes to the pathogenesis of chronic lung diseases (Demedts et al., 2006). Segura-Valdez et al. (2000) described an increase in endothelial cell apoptosis in lung tissue sections of patients with COPD compared to control patients. Although less frequent, alveolar epithelial cells, interstitial cells and inflammatory cells (neutrophils and lymphocytes) were also identified in the apoptosis process of these patients (Segura-Valdez et al., 2000; Funke et al., 2012). Epithelial cell apoptosis is also prominent in the bleomycin model of pulmonary fibrosis, in which intratracheal challenge leads to the rapid appearance of apoptosis in bronchial and alveolar epithelial cells (Funke et al., 2012). The method we used does not differentiate among cell types.

Pulmonary emphysema is generally characterized by an influx of inflammatory cells (neutrophils, macrophages, and CD8^+^ T lymphocytes) (Conese et al., 2013). We measured the levels of proinflammatory cytokines (TNF-α, IL-1β, IL-6, KC, MIP-2, and IFN-γ) and polymorphonuclear cells in lung tissue to assess the inflammatory profile triggered by intratracheal instillation of papain and the effect of BMMC. On the 14th day of the experimental protocol there was an increase in the levels of KC, MIP-2 and IFN-γ, as well as polymorphonuclear cell infiltration (P14 group, Table 1). BMMC therapy exhibited an anti-inflammatory action, reducing the levels of cytokines and the infiltration of polymorphonuclear cells (PC21 mice, Table 2). de Oliveira et al. (2017) observed in a murine model of pulmonary silicosis that BMMC (2 × 10^6^ cells/mice) were effective in returning to baseline levels the increased gene expression of some profibrotic and inflammatory cytokines. They suggest that the protection produced by cell treatment involves the suppression of inflammation as well as the production of regenerative growth factors. However, we did not find significant changes in the levels of TNF-α, IL-1β, and IL-6 in the pulmonary homogenate (Tables 1, 2).

In a murine model of elastase-induced pulmonary emphysema, cell therapy reduced the level of KC (mice IL-8 analog) and neutrophil infiltrate in lung tissue (Antunes et al., 2014). Using the same dose of BMMC as ours, Maron-Gutierrez et al. (2011) observed that the infiltrate of polymorphonuclear cells and mRNA expression of caspase-3, IL-1α, IL-1β, IL-1RN, and TGF-β decreased after BMMC therapy (2 × 10^6^ cells/animal) suggesting paracrine actions. Kurimoto et al. (2013) demonstrated elevated levels of KC, MIP-2, and IL-1β in the bronchoalveolar lavage fluid of C57BL/6 mice in the model of elastase-induced pulmonary emphysema at 21 days after exposure, in line with our findings. In mice, levels of TNF-α, IL-1β, and IL-6 show an early rise (peaking at 2–4 h after aggression) in a lipopolysaccharide-induced lung injury model (Faffe et al., 2000). Hence, after 4 days of elastase instillation TNF-α, IL-1β, and IL-6 are no longer present in the lung tissue (Vecchiola et al., 2011), supporting our results.

The lung target of injected stem cells is disputable. Mesenchymal cells (1–2 × 10^6^ cells/animal) were injected into the systemic circulation 5 days after lesion induction. After 30 days there was differentiation into type I pneumocytes. Migration and grafting of the transplanted cells may be related to the chemotactic factors released by the cellular epithelial lesion caused by bleomycin (Kotton et al., 2001). In addition to their proven plasticity, some authors argue that stem cells reach the pulmonary parenchyma soon after administration, modulate the damaging process, and then leave the lung tissue (Kim et al., 2014). Nonetheless, it is believed that the most beneficial effects of adult stem cell therapy result from their paracrine activity, i.e., their ability to modulate the synthesis of cytokines and growth factors without being present at the site of the lesion (Abreu et al., 2011; Conese et al., 2013; Kim et al., 2014).

We did not find any difference in peroxide concentration among groups. High levels of H_2_O_2_ promote differentiation, proliferation and migration of stem/progenitor cells. In response to tissue injury, the main sources of ROS, including H_2_O_2_, are NADPH oxidases. In some pathophysiological states, such as aging, atherosclerosis, heart failure, hypertension, diabetes, lung injury (Jin et al., 2015), excessive amounts of ROS create an inflammatory and oxidative microenvironment, inducing cellular damage and apoptosis of progenitor cells; the use of cells demonstrated a therapeutic effect on the regulation of the oxidative imbalance triggered by these diseases (Urao and Ushio-Fukai, 2013; Jin et al., 2015). Cell therapy showed an antioxidant effect on tissue injury induced by various causes, attenuating oxidative stress (Chen et al., 2011; Zhuo et al., 2011; Zhang et al., 2014; Jin et al., 2015). Possibly these antioxidant effects result from the paracrine and endocrine mechanisms of the cells to repair tissue damage (Bi et al., 2007; Xagorari et al., 2013). However, in the murine model of papain-induced pulmonary emphysema, the mechanisms of action as well as the participation of the NADPH oxidase pathway, including the DUOX enzymes in the pathogenesis of the disease, have not yet been described in the literature.

Type II alveolar cells express the enzymes DUOX 1 and DUOX 2, which, just as in the airways, are found in the apical pole of cells. Although DUOX 1 and 2 are found in the alveoli, there is scanty available information about their participation in H_2_O_2_ production at this histological level (Fischer, 2009). H_2_O_2_ production by these enzymes at the alveolar level is considered low in relation to the generation of H_2_O_2_ seen in the airway epithelium (Fischer et al., 2007). The precise role of the DUOXs enzymes in the pathogenesis of pulmonary emphysema is controversial and require more enlightening studies. We evaluated the mRNA expression for DUOX1 and DUOX2 and observed an increased expression of mRNA for DUOX1 and DUOX2 in the lung tissue (Figure 5) of the animals exposed to papain, in agreement with other groups (Ameziane-El-Hassani et al., 2005; Harper et al., 2005; Rigutto et al., 2009). Administration of 2 × 10^6^ BMMC attenuated mRNA expression for DUOX1 and DUOX2 in lung tissue. In addition, we observed an increase in the H_2_O_2_ generation and in the calcium-stimulated activity of DUOX in the lung tissue of animals treated with papain compared to the other groups, reinforcing the hypothesis that 2 × 10^6^ BMMC may behave as an antioxidant agent (Figure 6). There are distinct roles for the NADPH oxidase in the airways (Fischer, 2009). Despite the high structural similarity of the two DUOX isoforms, the production of H_2_O_2_ by DUOX2 is higher than by DUOX1 in the pulmonary airway epithelium (Ameziane-El-Hassani et al., 2005; Rigutto et al., 2009). However, normal airway epithelium expresses DUOX1 at higher levels than DUOX 2 (Schwarzer et al., 2004; Harper et al., 2005). Therefore, the release of H_2_O_2_ by DUOX1 and DUOX2 may be similar in the airways of normal individuals. Some cytokines selectively regulate DUOX1 and DUOX2 expression levels; IFN-γ positively regulates DUOX2 expression (Harper et al., 2005). We found that IFN-γ was augmented in papain-exposed mice and was reduced by BMMC (Table 2), in line with DUOX activity (Figure 6). Briefly, we emphasize the novelty of our results, since there is no description to date of the effects of cell therapy on the pathways of NADPH oxidases in a murine model of papain-induced pulmonary emphysema, specifically related to the regulation of DUOXs enzymes.

Our study presents limitations: (1) we did not measure the expression of NOX 1-5 enzymes in lung tissue after papain exposure; (2) we did not measure expression of growth factors and caspase-3; and (3) we did not measure collagen fibers.

In conclusion, 2 × 10^6^ BMMCs showed potent anti-inflammatory, antiapoptotic, antioxidant and restorative effects in papain-triggered pulmonary emphysema, possibly by blocking DUOX1 and reducing DUOX2.

## Author contributions

MNM, RF, MMM, and WZ: substantial contributions to the conception or design of the work; MNM, FM-R, NC, TM-G, VO, MMM, RF, and WZ; Substantial contributions to the acquisition, analysis, or interpretation of data for the work; MNM, FM-R, NC, TM-G, VO, MMM, RF, and WZ: drafting the work or revising it critically for important intellectual content; MNM, FM-R, NC, TM-G, VO, MMM, and WZ: final approval of the version to be published; MNM, FM-R, NC, TM-G, VO, MMM, RF, and WZ: agreement to be accountable for all aspects of the work in ensuring that questions related to the accuracy or integrity of any part of the work are appropriately investigated and resolved.

### Conflict of interest statement

The authors declare that the research was conducted in the absence of any commercial or financial relationships that could be construed as a potential conflict of interest.
